# Supplementary material for: Molecular self-assembly strategy tuning a dry crosslinking protein patch for biocompatible and biodegradable haemostatic sealing
Source: Nat Commun. 2025 Feb 7;16:1437. doi: 10.1038/s41467-025-56726-9 (PMC11806104; doi:10.1038/s41467-025-56726-9)
Supplement: Supplementary file 1 — Supplementary Information [file 41467_2025_56726_MOESM1_ESM.pdf]

Supplementary Information

Molecular self-assembly strategy tuning a dry crosslinking protein patch for biocompatible and biodegradable haemostatic sealing

Lisha Yu[1,2,#], Zhaodi Liu[1,2,#], Yong Zheng[3], Zongrui Tong[1,2], Yihang Ding[3], Weilin Wang[1,2,4,5,6,*], Yuan Ding[1,2,4,5,6,*], Zhengwei Mao[1,2,3,7,*]

[1] Department of Hepatobiliary and Pancreatic Surgery, The Second Affiliated Hospital, School of Medicine, Zhejiang University, Zhejiang, Hangzhou, 310009, China

[2] Key Laboratory of Precision Diagnosis and Treatment for Hepatobiliary and Pancreatic Tumor of Zhejiang Province, Zhejiang, Hangzhou, 310009, China

[3] MOE Key Laboratory of Macromolecular Synthesis and Functionalization, Department of Polymer Science and Engineering, Zhejiang University, Zhejiang, Hangzhou, 310058, China

[4] Research Center of Diagnosis and Treatment Technology for Hepatocellular Carcinoma of Zhejiang Province, Zhejiang, Hangzhou, 310009, China

[5] Center for Medical Research and Innovation in Digestive System Tumors, Ministry of Education, Zhejiang, Hangzhou, 310009, China

[6] Cancer Center, Zhejiang University, Zhejiang, Hangzhou, 310058, China

[7] State Key Laboratory of Transvascular Implantation Devices, Zhejiang, Hangzhou, 310009, China

[#] Lisha Yu and Zhaodi Liu contributed equally to this work.

*Correspondence and requests for materials should be addressed to Zhengwei Mao (zwmao@zju.edu.cn), Yuan Ding (dingyuan@zju.edu.cn) and Weilin Wang (wam@zju.edu.cn).

Supplementary Methods

Liquid chromatography-tandem mass spectrometry (LC-MS/MS)[1]: 100 μg of sample was dissolved in 200 μL of 5 M NH_4HCO_3. Then 2 μL of 0.5 μg/μL trypsin was added into sample solution, and incubated at 37 °C for 16 h for primary protein digestion. The sample solution was incubated with 4 μL of 1 M dithiothreitol (DTT) at 37 °C for 1 h. After that, the reaction was conducted at 25 °C for 30 min in dark with the addition of iodoacetamide (IAA, 7.4 mg mL^{-1}, 20 μL). The reaction was terminated by exposure to light for 30 min and dilatation to 8 mL. The second enzymatic digestion was further performed by trypsin (4 μL of 0.5 μg/μL trypsin) at 37 °C for 2 h. The peptides were separated using capillary column (nanoviper™,75 μm, i.d., 2 cm length) packed with C18 particles (3 μm, 100 Å) and capillary column (nanoviper™, 50 μm, i.d., 15 cm length) packed with C18 particles (2 μm, 100 Å). LTQ Orbitrap mass spectrometer (Thermo Fisher, Orbitrap Elite) was utilized for LC-MS/MS analysis. The sequences of human fibrinogen were obtained from UniProt (http://www.uniprot.org/). Peptides were processed using trypsin cleavage, and up to 5 missed cleavage sites were allowed. The amino acid sequence of the human fibrinogen was identified by using PEAKS Studio (version 8.0). Each group contains three independent samples.

Degree of hydrophobic substituents. Fluorescence method was used to determine the degree of hydrophobic substituents, owing to the ability of the unreacted lysine amines on the protein to convert the non-fluorescent fluorescamine into fluorescent product[2]. A fluorescamine solution (100 mg/mL) was freshly dissolved in dimethyl sulfoxide. A series of Fg solutions was prepared with a concentration range from 0 to 10 mg/mL. Then 1 μL of fluorescamine solution was added in to 100 μL of Fg solution, and kept in a dark place at 25 °C for 10 min to complete the derivative reaction. The fluorescence intensity was measured at 503 nm with excitation at 380 nm (Spark Multimode Microplate Reader). The standard curve of the primary amino concentration with fluorescence intensity was obtained. The fluorescence intensity of modified Fg was measured to qualitatively determine the degree of hydrophobic substituents. Each group contained three independent samples.

Mechanical tests (for double-sided adhesives)

The lap shear test was performed using the ASTM F2255-05 standard[3]. The prepared samples (10 mm × 20 mm) were added between the surfaces of two pieces of fresh porcine skin. After

10 min, two pieces of fresh porcine skin were then placed into a mechanical tester (Zwick/Roell Z020) with a constant tensile speed of 5 mm/min. The shear strength on the porcine skin was determined at the point of detachment. Each group contained three independent samples.

The T-peel test was performed using the ASTM F2256-05 standard[4]. The prepared sample (15 mm × 30 mm) was added between the surfaces of two pieces of fresh porcine skin. After 10 min, two pieces of fresh porcine skin were then placed into a mechanical tester (Zwick/Roell Z020) with a constant peeling speed of 5 mm/min. The measured force reached a plateau as the peeling process entered the steady state. Interfacial toughness was determined by dividing two times the plateau force (for 180-degree peel test) by the width of the tissue sample. Each group contained three independent samples.

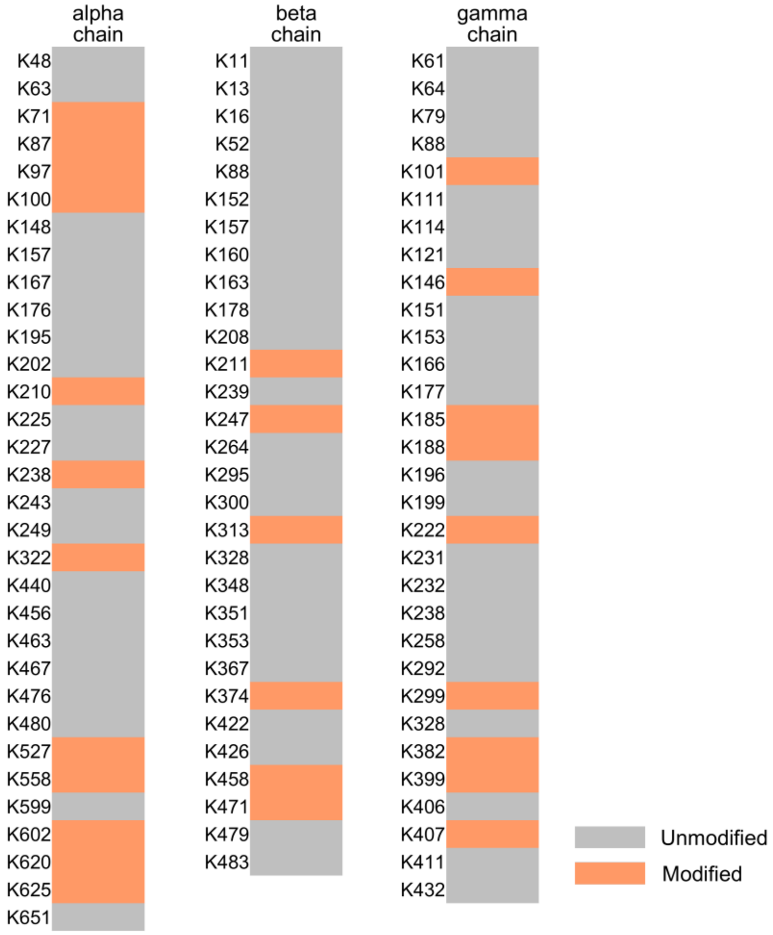

Supplementary Fig. 1. Lysine residues (K) with hydrophobic substituent of hexanoyl group (C6) analyzed using liquid chromatography-tandem mass spectrometry (LC-MS/MS). Fg is a dimeric protein composed of three polypeptide chains (alpha, beta, and gamma chains). The modified groups in three polypeptide chains were identified. The modified lysine residues were marked with orange color, and the unmodified lysine residues were marked with grey color. The primary amine of lysine residues was substituted with hexanoyl group (C6), and the degree of substitution was 21.6 ± 4.0%, n = 3 independent experiments (molar ratio of NHS-C6 to Fg: 54).

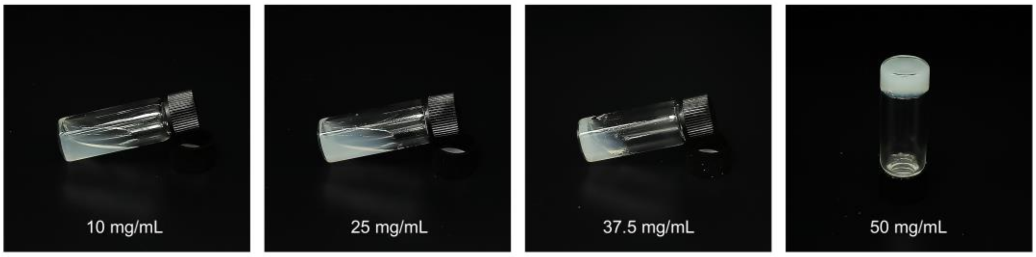

Supplementary Fig. 2. Photograph illustrating the gelation of FgC6 induced by hydrophobic interaction. When the concentration of FgC6 was 50 mg/mL, the hydrophobic interaction induces FgC6 gelation.

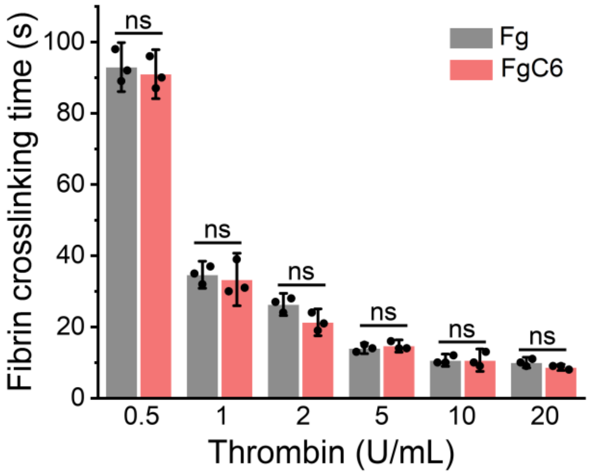

Supplementary Fig. 3. Crosslinking time of fibrin clot formation in the Fg and FgC6 systems (10 mg/mL). Fibrin clot formation is initiated by the thrombin-catalyzed release of FpA and FpB, which contributes to the conversion of fibrinogen to fibrin network. n = 3 independent samples. *P* values are determined by two-sided Student's t-test. Error bars, mean ± SD, ns: not significant.

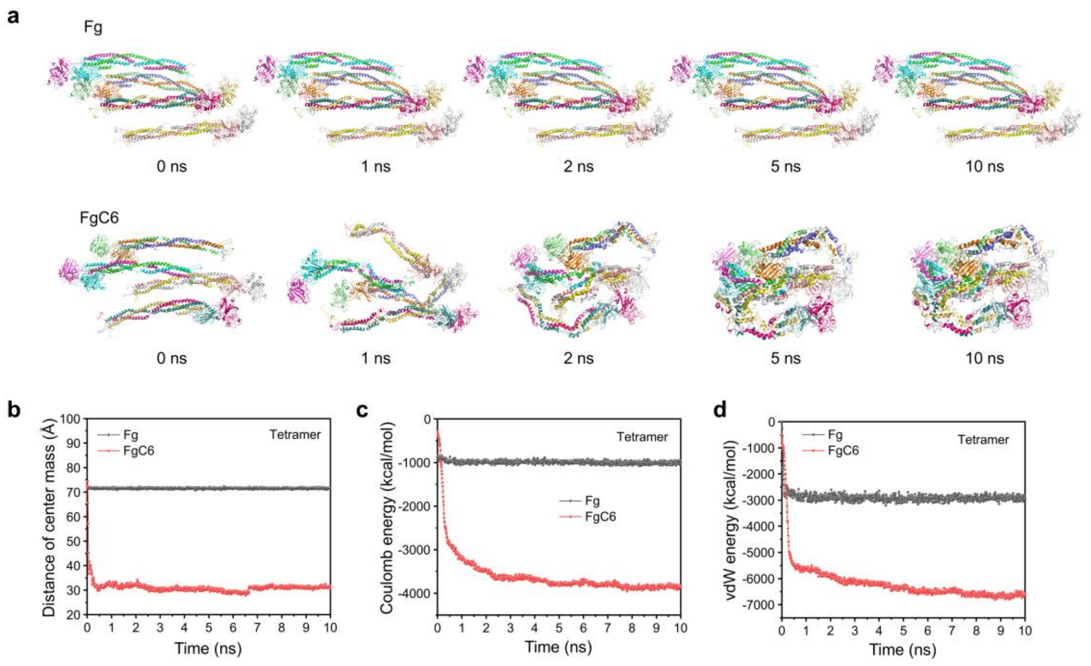

Supplementary Fig. 4. Intra/inter-molecular interaction and protein entanglement of FgC6 via MD simulation. a Conformation of Fg and FgC6 in the tetramer system during MD simulation. **b** Distance of center mass, **c** Coulomb energy, and **d** vdW energy of Fg and FgC6 in the tetramer system, respectively.

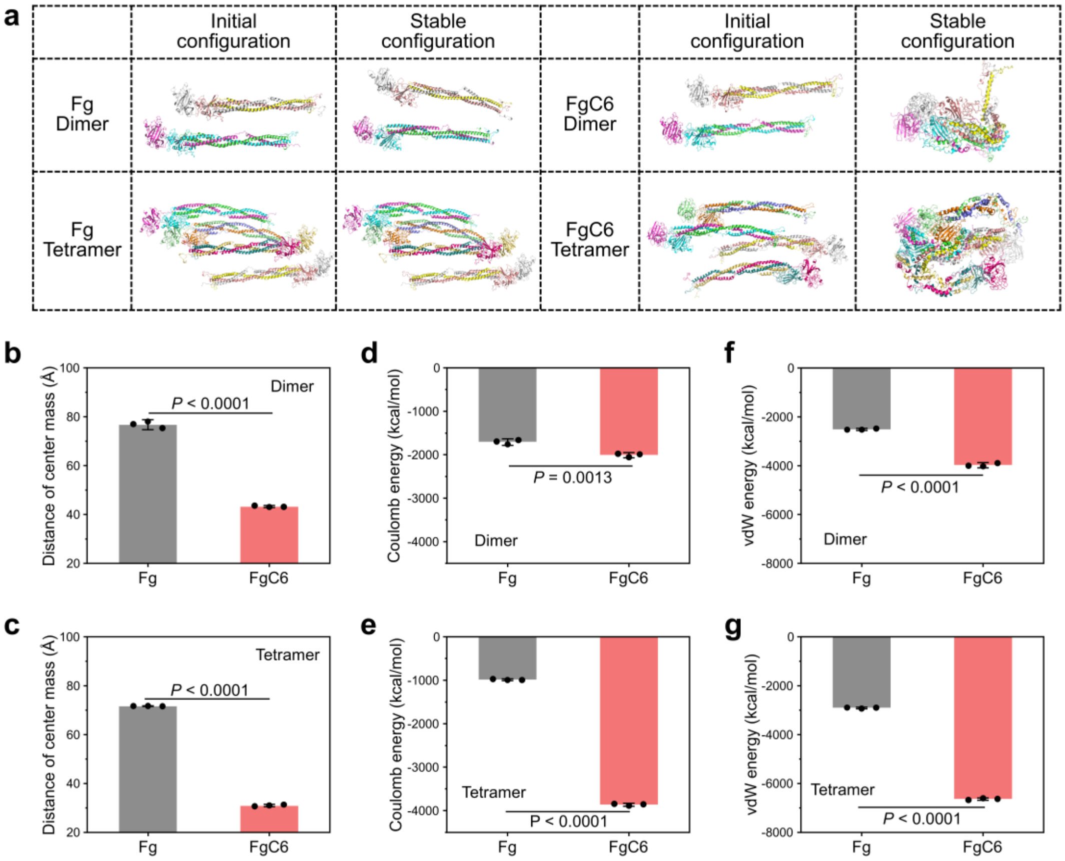

Supplementary Fig. 5. Intra/inter-molecular interaction and protein entanglement of FgC6 in the dimer and tetramer systems via MD simulation. a Initial and stable configuration of Fg and FgC6 in the dimer and tetramer systems, respectively. The FgC6 molecules with 20% substitution degree of lysine (20%Lys) were used for comparison between the dimer and tetramer systems. **b, c** Distance of center mass, **d, e** Coulomb energy, and **f, g** vdW energy of Fg and FgC6 molecules in the dimer and tetramer systems, respectively. n = 3 points at energy equilibration. *P* values are determined by a two-sided Student's t-test for **b-g**. Error bars, mean ± SD.

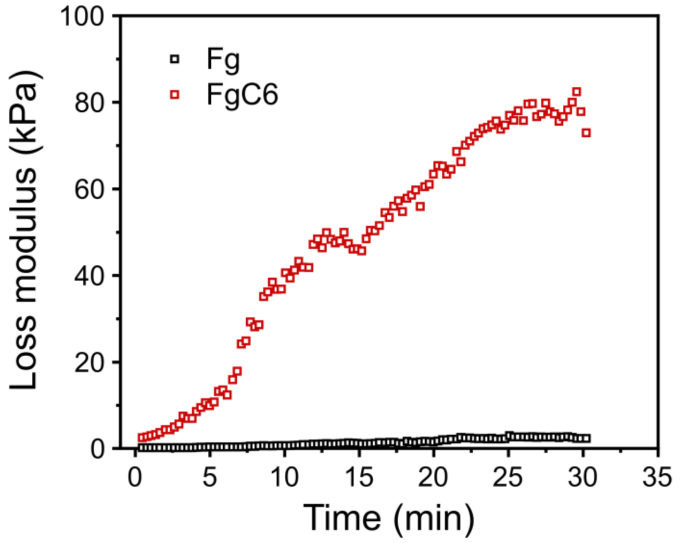

Supplementary Fig. 6. Mechanical property of FgC6 patch. Dynamic time-sweep rheological analysis of Fg and FgC6 for loss modulus G".

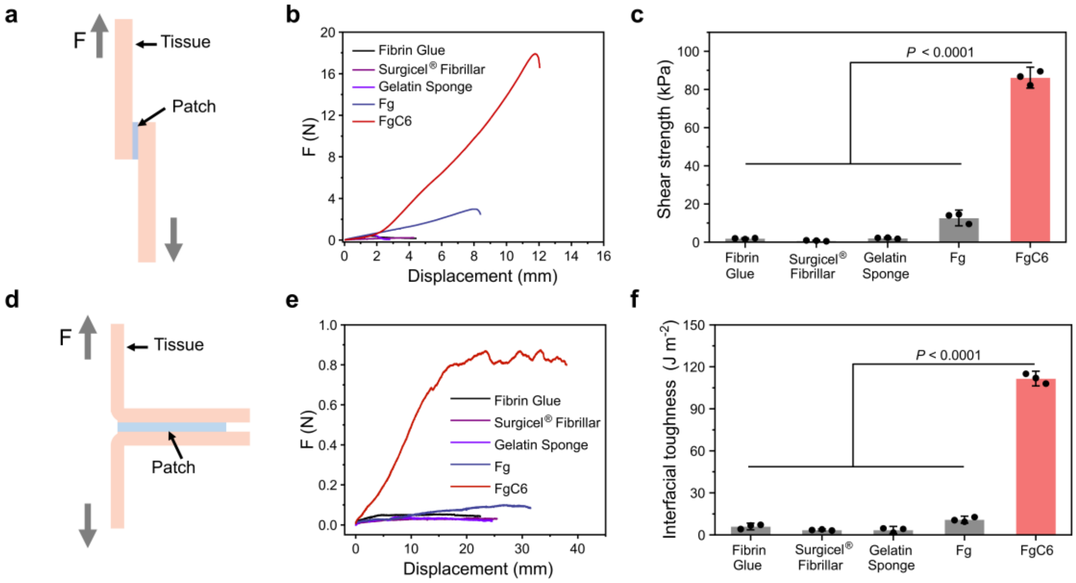

Supplementary Fig. 7. Adhesion performance of FgC6 patch. **a** Schematic of determining shear strength on the porcine skin based on standard lap-shear test (ASTM F2255-05). **b** Force–displacement curves of the lap shear test. **c** Shear strength of Fibrin Glue, Surgicel® Fibrillar, Gelatin Sponge, Fg and FgC6, respectively. **d** Schematic of interfacial toughness on the porcine skin based on the standard T-peel test (ASTM F2256-05). **e** Force–displacement curves of the T-peel adhesion test. **f** Interfacial toughness of Fibrin Glue, Surgicel® Fibrillar, Gelatin Sponge, Fg and FgC6, respectively. n = 3 independent samples for **c** and **f**. *P* values are determined by one-way ANOVA followed by Tukey's post-hoc test for **c** and **f**. Error bars, mean ± SD.

Supplementary Table 1. Hydrophobic substituents of hexanoyl group (C6) on FgC6

		Molar ratio of NHS-C6 to Fg	Substitution rate [a]
1	FgC6-1	14	4.0 ± 2.7%
2	FgC6-2	27	8.4 ± 2.1%
3	FgC6-3	54	20.7 ± 1.7%
4	FgC6-4	82	30.5 ± 1.6%

[a] Substitution rate of hexanoyl group (C6) was analyzed using the fluorescence method with fluorescamine. Mean ± SD, n = 3 independent samples.

Supplementary Table 2. Adhesion performance and distance of center mass of protein molecules in FgC6 patches

		Substitution rate	Distance of center mass (Å)[a]	Shear strength (kPa)[b]
1	Fg	0	76.8 ± 1.4	12.7 ± 2.8
2	FgC6-1	4.0 ± 2.7%	-	24.2 ± 0.8
3	FgC6-2	8.4 ± 2.1%	68.9 ± 0.1	57.0 ± 1.7
4	FgC6-3	20.7 ± 1.7%	43.2 ± 1.5	86.2 ± 3.7
5	FgC6-4	30.5 ± 1.6%	-	107.6 ± 15.8

[a] The distance of center mass between two protein molecules was obtained from MD simulation.
[b] The shear strength on porcine skin was investigated based on standard lap-shear test (ASTM F2255-05). Mean ± SD, n = 3 independent samples.

Supplementary Table 3. Adhesion performance of protein hydrogel patches

Modification type	Sample [a]	Modified group [b]		Shear strength (kPa) [c]
Natural protein	Fg	-		12.7 ± 2.8
Hydrophobic group modification	FgC6	hexanoyl	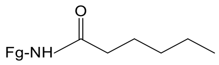	86.2 ± 3.7
	FgC2	acetyl	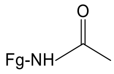	30.5 ± 3.6
	FgC3	propionyl	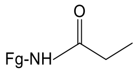	41.5 ± 1.7
	FgC10	decanoyl	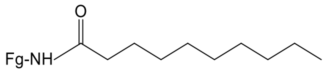	96.4 ± 4.5
	FgC12	dodecanoyl	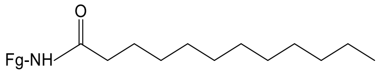	110.6 ± 3.1
	Fg-Ben	benzoyl	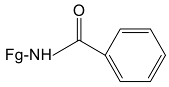	85.8 ± 7.6
	Fg-Nap	2-(naphthalene-1-yl)acetyl	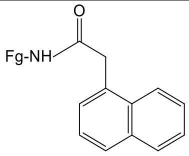	120.0 ± 3.5
Hydrophilic group modification	Fg-C10O4	2,5,8,11-tetraoxatetradecane-14-acyl	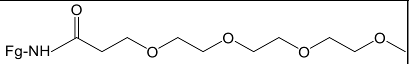	6.8 ± 1.0
	Fg-C9COOH	10-oxocapric acid acyl	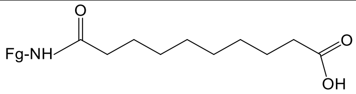	11.8 ± 3.5
Other cross-linking methods	Chemical cross-linking	Methacryloyl [d], light cross-linking	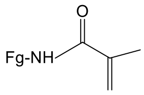	12.5 ± 1.1
	Enzymatic cross-linking	Thrombin mediated cross-linking	Converting fibrinogen to fibrin network	8.5 ± 0.8

[a] Four fibrinogen-based patches (hydrophobic group modification, hydrophilic group modification, chemical cross-linking method, enzymatic cross-linking) were prepared using the same source of fibrinogen to compare their adhesion performance. The patches of hydrophobic group modification and hydrophilic group modification were prepared using a molar ratio of 54 of NHS-Cn to Fg. Other cross-linking methods have the same content of fibrinogen or modified fibrinogen as the FgC6 patch.

[b] The modified groups were using NHS-Cn to modify the lysine residues of Fg.

[c] The shear strength was measured based on standard lap-shear test (ASTM F2255-05). Mean ± SD. n = 3.

[d] The substitution degree of methacryloyl group for chemical cross-linking patch (21.2± 0.4%) was comparable to that of C6 group for FgC6 (20.7 ± 1.7%). Mean ± SD, n = 3 independent samples.

Supplementary Table 4. Bleeding outcomes following injury and treatment in porcine liver injury model

	Pretreatment blood loss (g) [a]	Posttreatment blood loss (g) [b]	Haemostatic time (s)
Surgicel® Fibrillar	22.0 ± 4.6	38.5 ± 6.6	258 ± 27
Surgiflo®(Thrombin)	22.9 ± 3.0	9.2 ± 3.8	180 ± 45
TachoSil®	22.2 ± 3.7	5.0 ± 1.3	204 ± 33
FgC6	21.5 ± 4.7	1.9 ± 0.9	30 ± 0
P (among groups) [c]	0.9575	< 0.0001	< 0.0001

[a] Free bleeding within 30 s was recorded.

[b] Blood loss following treatment was recorded until successful haemostasis.

[c] *P* values are determined by one-way ANOVA. Mean ± SD, n = 5 independent injuries.

Supplementary Table 5. Bleeding outcomes following injury and treatment in porcine femoral artery injury model

	Pretreatment blood loss (g) [a]	Posttreatment blood loss (g) [b]	Haemostatic time (s)
Combat Gauze®	175.5 ± 39.1	22.0 ± 6.5	330 ± 83
FgC6	188.0 ± 30.9	0.4 ± 0.2	60 ± 0
P (among groups) [c]	0.5548	< 0.0001	< 0.0001

[a] Free bleeding within 45 s was recorded.

[b] Blood loss following treatment was recorded until successful haemostasis.

[c] *P* values are determined by two-sided Student’s t-test. Mean ± SD, n = 6 independent injuries.

Supplementary Table 6. Comparison of commercially available haemostatic materials

**Haemostatic material**	**Product Name**	**Major Components** [a]	**Advantages** [a]
Fibrinogen patch	FgC6 patch<br>(this study)	Fibrinogen patch via protein molecular self-assembly strategy: fibrinogen modified with hydrophobic groups	— Robust adhesion<br>— Biocompatibility<br>— Biodegradability<br>— No preparation required
Fibrin sealant patch	EVARREST®	Fibrinogen-thrombin-embedded oxidized cellulose patch: oxidized regenerated cellulose and polygactin 910 non-woven fibers (carrier), fibrinogen and thrombin (active ingredient)	— Biocompatibility<br>— Biodegradability<br>— No preparation required
Fibrin sealant patch	TachoSil®	Fibrinogen-thrombin-impregnated collagen patch: collagen (carrier), fibrinogen and thrombin (active ingredient)	— Biocompatibility<br>— Biodegradability<br>— No preparation required
Fibrin sealant	Fibrin Glue<br>(Tisseel®, Evicel®,<br>Artiss®, RAAS®)	Fibrinogen, thrombin	— Biocompatibility<br>— Biodegradability<br>— Fast gelation
Oxidized cellulose dressing	Surgicel® Fibrillar	Oxidized regenerated cellulose	— Bactericidal property<br>— Biocompatibility
Gelatin Sponge	Gelfoam®, Gelfilm®, Xiangen®	Gelatin	— Biodegradability<br>— Low immunogenicity

Gelatin matrix	Surgiflo®	Gelatin matrix	— Providing tamponade effect<br>— Low immunogenicity
Gelatin matrix with thrombin	Surgiflo®(Thrombin)	Gelatin matrix, thrombin (active ingredient)	— Providing tamponade effect<br>— Low immunogenicity
Gelatin matrix with thrombin	Floseal®	Gelatin matrix, thrombin (active ingredient)	— Providing tamponade effect<br>— Low immunogenicity
Mineral kaolin dressing	Combat Gauze®	Non-woven gauze (carrier), kaolin (active ingredient)	— Procoagulant activity<br>— Low cost<br>— Good storability

[a] The major components and advantages of haemostatic materials were mainly obtained from their package inserts.

Supplementary Table 7. Methods of application for commercial products from manufacturer's guidelines

Product Name	Dosage and administration	Methods of application	Limitations for use/Contraindications	Reference
TachoSil® Fibrin sealant patch	--TachoSil is an adjunct to haemostasis in cardiovascular and hepatic surgery, when control of bleeding by standard surgical techniques (such as suture, ligature or cautery) is ineffective or impractical.<br><br>--TachoSil is applied to the surface of cardiac, vascular or hepatic tissue only.	(1) Determine the number of patches to be applied by the size of the bleeding area.<br>(2) Select the appropriate TachoSil patch so that it extends 1 to 2 cm beyond the margins of the wound.<br>(3) cleanse the area to be treated.<br>(4) disinfectants and other fluids hold in place with gentle pressure applied through moistened gloves or a moist pad for at least 3 min.<br>(5) To avoid pulling the patch loose, first place a clean surgical instrument at one end of the patch before relieving the pressure	--Not for use in place of sutures or other forms of mechanical ligation in treatment of major arterial or venous bleeding.<br><br>--Do not apply TachoSil intravascularly. Intravascular application of TachoSil may result in life-threatening thromboembolic events.	Package Insert---TachoSil [2024]
Surgicel® Fibrillar	--Surgicel Fibrillar is used adjunctively in surgical procedures to assist in the control of capillary, venous and small arterial hemorrhage when ligation or other conventional methods of control are impractical or ineffective.	Use only as much Surgicel Fibrillar as is necessary for haemostasis, holding it firmly in place until bleeding stops.	--Surgicel Fibrillar should not be used to control hemorrhage from large arteries.	Package-Surgicel-[2021]
Surgiflo® (Thrombin)	--Surgiflo, mixed with thrombin solution, is indicated in surgical procedures (other than ophthalmic) as an adjunct to haemostasis when control of bleeding by ligature or other conventional methods is ineffective or impractical.	(1) Identify the source of bleeding.<br>(2) Surgiflo can be used with or without the applicator tip attached to the syringe. Apply sufficient Surgiflo Haemostatic Matrix to cover the entire bleeding surface.<br>(3) Apply a sterile saline moistened gauze over the Surgiflo to ensure the material	--Do not use Surgiflo in intravascular compartments because of the risk of embolization.<br>--Do not use Surgiflo in closure of skin incisions because it may interfere with the healing of skin edges.<br>--Surgiflo should not be used in instances	Package- Surgiflo [2020]

		remains in contact with the bleeding tissue.<br>(4) After 1-2 min, lift the gauze and inspect the wound site. Once bleeding has ceased, irrigate excess Surgiflo away gently so as not to disturb the new clot.	of pumping arterial hemorrhage. It should not be used where blood or other fluids have pooled or in cases where the point of hemorrhage is submerged.	
Combat Gauze®	--Penetrating wounds and massive bleeding<br>--Gunshot wounds<br>--Stabbings<br>--Large non-compressible injuries where tourniquets cannot be applied	(1) Pack Combat Gauze into wound and use it to apply pressure directly over bleeding source<br>(2) Continue to apply pressure for 3 min or until bleeding stops<br>(3) Wrap and tie bandage to maintain pressure. Seek medical care immediately.	--For temporary external use to control traumatic bleeding	Package--Combat Gauze-[2023]

Supplementary Table 8. Applications of haemostatic materials in porcine liver injury model

**Product Name**	**Applications of haemostatic materials on the injury**	**How haemostasis was confirmed**
FgC6 patch (this study)	Directly applied on the wound with manual compression for 30 s.	The compression was interrupted after 0.5, 1 min to check for the haemostasis.
TachoSil® Fibrin sealant patch	Applied on the wound with manual compression through a moist pad for at least 3 min.	The compression was interrupted after 3, 4, 5, 8 min to check for the haemostasis.
Surgicel® Fibrillar	Hold firmly on the wound for at least 3 min.	The compression was interrupted after 3, 4, 5, 8 min to check for the haemostasis.
Surgiflo®(Thrombin)	Cover the entire bleeding surface and apply a moist pad over the Surgiflo® for at least 2 min.	After 2, 3, 4, 5, 8 min, lift the moist pad to check for the haemostasis.

Supplementary Table 9. Applications of haemostatic materials in porcine femoral artery injury model

**Product Name**	**Applications of haemostatic materials on the injury**	**How haemostasis was confirmed**
FgC6 patch (this study)	Applied on the wound with through a moist pad for 1 min.	The compression was interrupted after 1, 2 min to check for the haemostasis. The survival of injured pigs was observed within 3 h.
Combat Gauze®	Applied on the wound for at least 3 min.	The compression was interrupted after 3, 4, 5, 8 min to check for the haemostasis. The survival of injured pigs was observed within 3 h.

Supplementary References

1 Zhou, Y. *et al.* Prediction of ligand modulation patterns on membrane receptors via lysine reactivity profiling. *Chem. Commun.* **55**, 4311-4314, (2019).

2 Yu, N. *et al.* Preparation and properties of cationic gelatin cross-linked with tannin. *J. Agric. Food Chem.* **68**, 9537-9545, (2020).

3 Wu, J. *et al.* An off-the-shelf bioadhesive patch for sutureless repair of gastrointestinal defects. *Sci. Transl. Med.* **14**, eabh2857, (2022).

4 Yuk, H. *et al.* Rapid and coagulation-independent haemostatic sealing by a paste inspired by barnacle glue. *Nat. Biomed. Eng.* **5**, 1131-1142, (2021).
