# Supplementary material for: Molecular self-assembly strategy tuning a dry crosslinking protein patch for biocompatible and biodegradable haemostatic sealing
Source: Nat Commun. 2025 Feb 7;16:1437. doi: 10.1038/s41467-025-56726-9 (PMC11806104; doi:10.1038/s41467-025-56726-9)
Supplement: Supplementary file 2 — Description of Additional Supplementary Files [file 41467_2025_56726_MOESM2_ESM.pdf]

Description of Additional Supplementary Files

Supplementary Movie 1. In vivo haemostatic performance of Surgicel® Fibrillar in porcine liver injury model.

Supplementary Movie 2. In vivo haemostatic performance of Surgiflo®(Thrombin) in porcine liver injury model.

Supplementary Movie 3. In vivo haemostatic performance of TachoSil® in porcine liver injury model.

Supplementary Movie 4. In vivo haemostatic performance of FgC6 patch in porcine liver injury model.

Supplementary Movie 5. In vivo haemostatic performance of Combat Gauze® in porcine femoral artery injury model.

Supplementary Movie 6. In vivo haemostatic performance of FgC6 patch in porcine femoral artery injury model.
