# Supplementary material for: Molecular self-assembly strategy tuning a dry crosslinking protein patch for biocompatible and biodegradable haemostatic sealing
Source: Nat Commun. 2025 Feb 7;16:1437. doi: 10.1038/s41467-025-56726-9 (PMC11806104; doi:10.1038/s41467-025-56726-9)
Supplement: Supplementary file 9 — Reporting Summary [file 41467_2025_56726_MOESM9_ESM.pdf]

nature portfolio

Corresponding author(s): Zhengwei Mao, Yuan Ding, Weilin Wang

Last updated by author(s): Jan 20, 2025

Reporting Summary

Nature Portfolio wishes to improve the reproducibility of the work that we publish. This form provides structure for consistency and transparency in reporting. For further information on Nature Portfolio policies, see our Editorial Policies and the Editorial Policy Checklist.

Statistics

For all statistical analyses, confirm that the following items are present in the figure legend, table legend, main text, or Methods section.

n/a	Confirmed	
☐	☒	The exact sample size (*n*) for each experimental group/condition, given as a discrete number and unit of measurement
☐	☒	A statement on whether measurements were taken from distinct samples or whether the same sample was measured repeatedly
☐	☒	The statistical test(s) used AND whether they are one- or two-sided<br>*Only common tests should be described solely by name; describe more complex techniques in the Methods section.*
☒	☐	A description of all covariates tested
☐	☒	A description of any assumptions or corrections, such as tests of normality and adjustment for multiple comparisons
☐	☒	A full description of the statistical parameters including central tendency (e.g. means) or other basic estimates (e.g. regression coefficient) AND variation (e.g. standard deviation) or associated estimates of uncertainty (e.g. confidence intervals)
☐	☒	For null hypothesis testing, the test statistic (e.g. *F*, *t*, *r*) with confidence intervals, effect sizes, degrees of freedom and *P* value noted<br>*Give P values as exact values whenever suitable.*
☒	☐	For Bayesian analysis, information on the choice of priors and Markov chain Monte Carlo settings
☒	☐	For hierarchical and complex designs, identification of the appropriate level for tests and full reporting of outcomes
☒	☐	Estimates of effect sizes (e.g. Cohen's *d*, Pearson's *r*), indicating how they were calculated

Our web collection on statistics for biologists contains articles on many of the points above.

Software and code

Policy information about availability of computer code

Data collection	1. Cryogenic transmission electron microscopy (Cryo-TEM) was taken with FEI Talos F200C transmission electron microscopy (200k).<br>2. Synchrotron small-angle X-ray scattering (SAXS) measurements were conducted on the BL19U2 beamline at Shanghai Synchrotron Radiation Facility.<br>3. Secondary structure of protein system was characterized by FT-IR spectroscopy (Thermo Scientific Nicolet iS20).<br>4. Rheological measurements were carried out using a rotary rheometer (HAAKE RS6000).<br>5. Mechanical tests were conducted on a mechanical tester (Zwick/Roell Z020).<br>6. Fluorescence images for cell viability were acquired using a fluorescence microscope (KEYENCE).<br>7. Hematoxylin and eosin (H&E) staining images were acquired using a Digital Slide Scanner (HAMAMATSU PHOTONICS, NanoZoomer®S360).<br>8. Immunofluorescence images were acquired using a digital microscopy (3DHistech, Pannoramic MIDI).<br>9. Liquid chromatography-tandem mass spectrometry (LC-MS/MS) was taken with LTQ Orbitrap mass spectrometer (Thermo Fisher, Orbitrap Elite).<br>10. Fluorescence intensity was measured on Spark Multimode Microplate Reader.<br>The software used for data collection was described in “Methods” section and Supplementary Methods. No special code was used for data collection.
Data analysis	1. Statistical analysis was performed using OriginLab (version 2019b).<br>2. Synchrotron small-angle X-ray scattering (SAXS) raw data were analyzed by FIT2D (version 10.132).<br>3. MD simulation was performed in GROMACS software (version 5.1.5).<br>4. Hematoxylin and eosin (H&E) staining images were visualized by NDP.view (version 2.9.22).<br>5. Immunofluorescence images were visualized by CaseViewer (version 2.4).

6. Fluorescence images were analyzed by using ImageJ (version 2.9.0).
7. Amino acid sequence of fibrinogen in LC-MS/MS was identified by using PEAKS Studio (version 8.0).

For manuscripts utilizing custom algorithms or software that are central to the research but not yet described in published literature, software must be made available to editors and reviewers. We strongly encourage code deposition in a community repository (e.g. GitHub). See the Nature Portfolio guidelines for submitting code & software for further information.

Data

Policy information about availability of data

All manuscripts must include a data availability statement. This statement should provide the following information, where applicable:

- Accession codes, unique identifiers, or web links for publicly available datasets
- A description of any restrictions on data availability
- For clinical datasets or third party data, please ensure that the statement adheres to our policy

All data needed to support the findings of this study are provided within the article, Supplementary information, and Source data file. This study utilizes publicly accessible data from the Protein Data Bank (PDB) under accession code: 3GHG. Source data are provided with this paper. Source data is available for Figures 1-6, and Supplementary Figure 3, 4, 5 ,6 and 7 in the associated source data file.

Research involving human participants, their data, or biological material

Policy information about studies with human participants or human data. See also policy information about sex, gender (identity/presentation), and sexual orientation and race, ethnicity and racism.

Reporting on sex and gender	N/A
Reporting on race, ethnicity, or other socially relevant groupings	N/A
Population characteristics	N/A
Recruitment	N/A
Ethics oversight	N/A

Note that full information on the approval of the study protocol must also be provided in the manuscript.

Field-specific reporting

Please select the one below that is the best fit for your research. If you are not sure, read the appropriate sections before making your selection.

☒ Life sciences ☐ Behavioural & social sciences ☐ Ecological, evolutionary & environmental sciences

For a reference copy of the document with all sections, see nature.com/documents/nr-reporting-summary-flat.pdf

Life sciences study design

All studies must disclose on these points even when the disclosure is negative.

Sample size	No statistical method was used to predetermine the sample size for each study. In order to ensure reproducibility and statistical significance, sample sizes were based on previously published studies assessing similar events (PMID: 34373600; PMID: 36028516). For property measurement experiments, samples were prepared and tested at least twice. For in vitro and in vivo studies, each group contains at least three independent experiments. The number of the independent experiments was indicated in each figure legend and Methods section.
Data exclusions	No data was excluded from the analysis.
Replication	All the findings were reliably reproduced in multiple independent experiments. For property measurement experiments, our data represent at least two independent assays that produce similar results. We have used different assays to confirm our findings. For in vitro and in vivo studies, each group contains at least three independent experiments with similar results.
Randomization	For the characterizations and adhesion performance experiments, there is no need to allocate. For the animal experiments, animals used were randomly assigned to each treatment group. For the cell experiments, cells were randomly allocated to experimental groups.
Blinding	The investigators were blinded to group allocation during data collection in the animal experiments. The hemostatic time and blood loss were collected blindly. For histological analyses, slides were binded prior to scoring.

Reporting for specific materials, systems and methods

We require information from authors about some types of materials, experimental systems and methods used in many studies. Here, indicate whether each material, system or method listed is relevant to your study. If you are not sure if a list item applies to your research, read the appropriate section before selecting a response.

Materials & experimental systems

n/a	Involved in the study	
☐	☒	Antibodies
☐	☒	Eukaryotic cell lines
☒	☐	Palaeontology and archaeology
☐	☒	Animals and other organisms
☒	☐	Clinical data
☒	☐	Dual use research of concern
☒	☐	Plants

Methods

n/a	Involved in the study	
☒	☐	ChIP-seq
☒	☐	Flow cytometry
☒	☐	MRI-based neuroimaging

Antibodies

Antibodies used	All antibodies have been described as required in Methods section.<br>Primary antibodies:<br>Rabbit multiclonal antibody to anti-CD 68 (Abcam, ab303565, 1:400 for Immunofluorescence);<br>Rabbit monoclonal antibody to anti-CD 206 (Cell Signaling Technology, #24595, 1:200 for Immunofluorescence);<br>Secondary antibody:<br>Alexa Fluor 488 labeled anti-rabbit secondary antibody (Life technologies, A21206, 1:400 for Immunofluorescence).
Validation	All antibodies used in our study have been validated and detailed information could be found on the website from manufactures as listed below. Additional information on validation can be found on the manufacture's websites.<br>1. Rabbit multiclonal antibody to anti-CD 68 (Abcam, ab303565): Manufacturer validated that this multiclonal antibody recognizes mouse, rat and human CD 68. Application: IHC-P, IHC-Fr, ICC/IF, WB. https://www.abcam.cn/products/primary-antibodies/cd68-antibody-rm1031-ab303565<br>2. Rabbit monoclonal antibody to anti-CD 206 (Cell Signaling Technology, #24595): Manufacturer validated that this monoclonal antibody recognizes human, mouse, rat and monkey CD 206. Application: IP, IHC-P, ICC/IF, WB. https://www.cellsignal.cn/products/primary-antibodies/cd206-mrc1-e6t5j-xp-rabbit-mab/24595

Eukaryotic cell lines

Policy information about cell lines and Sex and Gender in Research

Cell line source(s)	L929 cell lines were obtained from the American Type Culture Collection (ATCC, CCL-1).
Authentication	Cell lines from ATCC are routinely authenticated by cellular morphology assay.
Mycoplasma contamination	We confirm that the cell lines were negative for mycoplasma contamination.
Commonly misidentified lines (See ICLAC register)	No commonly misidentified cell lines were used in this study.

Animals and other research organisms

Policy information about studies involving animals; ARRIVE guidelines recommended for reporting animal research, and Sex and Gender in Research

Laboratory animals	Sprague Dawley rats (male, 8 weeks, 250-300 g) and Bama pigs (female, 22-26 kg) were used for in vivo studies.
Wild animals	The study did not involve wild animals.
Reporting on sex	The sex of the animals used in this study was provided in the methods. For the experiments with Sprague Dawley rats, male rats were used. For the experiments with Bama pigs, female pigs were used. Sex was not considered in the study design.
Field-collected samples	The study did not involve samples collected from the field.
Ethics oversight	In vivo study protocols were approved by the Laboratory Animal Welfare and Ethics Committee of Zhejiang University (ZJU20220337) and The Second Affiliated Hospital of Zhejiang University School of Medicine (SAHZU2024#248).

Note that full information on the approval of the study protocol must also be provided in the manuscript.
